# Short DNA sequence patterns accurately identify broadly active human enhancers

**DOI:** 10.1186/s12864-017-3934-9

**Published:** 2017-07-17

**Authors:** Laura L. Colbran, Ling Chen, John A. Capra

**Affiliations:** 1 Vanderbilt Genetics Institute, Vanderbilt University, Nashville, TN 37235 USA; 2 Department of Biological Sciences, Vanderbilt University, Nashville, TN 37235 USA; 3 Center for Structural Biology, Departments of Biomedical Informatics and Computer Science, Vanderbilt University, Nashville, TN 37235 USA

**Keywords:** Enhancer, Dinucleotide repeat motif, Machine learning, Gene expression

## Abstract

**Background:**

Enhancers are DNA regulatory elements that influence gene expression. There is substantial diversity in enhancers’ activity patterns: some enhancers drive expression in a single cellular context, while others are active across many. Sequence characteristics, such as transcription factor (TF) binding motifs, influence the activity patterns of regulatory sequences; however, the regulatory logic through which specific sequences drive enhancer activity patterns is poorly understood. Recent analysis of *Drosophila* enhancers suggested that short dinucleotide repeat motifs (DRMs) are general enhancer sequence features that drive broad regulatory activity. However, it is not known whether the regulatory role of DRMs is conserved across species.

**Results:**

We performed a comprehensive analysis of the relationship between short DNA sequence patterns, including DRMs, and human enhancer activity in 38,538 enhancers across 411 different contexts. In a machine-learning framework, the occurrence patterns of short sequence motifs accurately predicted broadly active human enhancers. However, DRMs alone were weakly predictive of broad enhancer activity in humans and showed different enrichment patterns than in *Drosophila*. In general, GC-rich sequence motifs were significantly associated with broad enhancer activity, and consistent with this enrichment, broadly active human TFs recognize GC-rich motifs.

**Conclusions:**

Our results reveal the importance of specific sequence motifs in broadly active human enhancers, demonstrate the lack of evolutionary conservation of the role of DRMs, and provide a computational framework for investigating the logic of enhancer sequences.

**Electronic supplementary material:**

The online version of this article (doi:10.1186/s12864-017-3934-9) contains supplementary material, which is available to authorized users.

## Background

Enhancers are DNA regulatory elements distal to promoters that bind transcription factors (TFs) to drive tissue-specific gene expression. They control patterns of gene expression during development, allowing diverse tissues to differentiate from a single cell and continue functioning properly in maturity [1, 2]. Because enhancers play a central role in regulating essential transcriptional programs, genome-wide association studies (GWAS) often implicate non-coding variation in enhancer regions as associated with risk for numerous complex diseases [3, 4]. Several in-depth experimental analyses of loci identified by GWAS have revealed that the causal mutations in these regions disrupt enhancer activity [5–8]. However, the function of many of these variants is unknown, and it can be unclear in what cell types they alter activity. Better understanding of how enhancer sequences drive activity patterns across cellular contexts would enable more accurate interpretation of the effects of non-coding mutations.

Enhancers harbor binding motifs recognized by TFs; thus, the information encoded in enhancer sequences provides valuable information about regulatory specificity [2, 9]. Technological advances in high-throughput sequencing have enabled the development of genome-scale assays to identify sequences with putative enhancer activity. Several large-scale efforts have applied methods such as chromatin immunoprecipitation followed by sequencing (ChIP-seq) [10], identification of DNaseI-hypersensitive sites (DHS) via sequencing (DNase-seq) [11], and identification of enhancer RNA (eRNA) transcription via cap analysis of gene expression (CAGE) [12] to map putative enhancers over many tissues and cell lines [13–16].

Analyses of these and smaller-scale enhancer datasets have enabled identification of the unique sequence and chromatin properties of enhancers active in different tissues, which can then be used to predict enhancers in other contexts [17–19]. Indeed, enhancer-finding algorithms based solely on sequence information have successfully predicted active enhancers in many tissues [20–24]. These algorithms usually perform better than enhancer-finding algorithms built only on the occurrence profiles of known TF motifs, suggesting that the algorithms detect previously unidentified functional sequence characteristics that, if interpreted, could fill gaps in current knowledge about TF binding specificities and other enhancer sequence properties. For example, a recent study proposed a model in which short repetitive sequences—dinucleotide repeat motifs (DRMs)—promote general enhancer activity and play an essential role in driving broad enhancer activity across many cell types [16]. In spite of these successes, we still lack a comprehensive understanding of how enhancer sequences drive their activity across tissues and development.

In this study, we comprehensively analyzed the of ability of short DNA sequence patterns, including DRMs, to predict the breadth of activity of tens of thousands of human enhancers across hundreds of human tissues. First, we computed the enrichment of DRMs among broadly active enhancers, and unlike in *Drosophila*, we consistently observed significant enrichment of GC DRMs and depletion of TA DRMs. To evaluate the ability of DRMs to predict broadly active enhancers, we trained a support vector machine (SVM) classification algorithm on the occurrence patterns of DRMs. In further contrast to results in *Drosophila,* we found that DRMs alone were only weakly predictive of broadly active enhancers versus context-specific enhancers or random regions from the genomic background. However, when trained on all possible 6-bp sequences, SVMs could readily distinguish between broadly active, context-specific, and genomic background regions. The 6-mer sequence patterns most enriched—and most predictive—of broadly active enhancers were GC-rich, suggesting that DRM contributions to enhancer activity are part of a larger trend seen among other 6-mers that is driven by GC content. Furthermore, we show that broadly active human TFs are more likely to bind GC-rich sequences than tissue-specific TFs. Thus, we conclude that DRMs are not unique drivers of human enhancer activity, but broadly active human enhancers exhibit distinct sequence properties.

## Methods

### Enhancer data

We focused our analyses on enhancers identified by CAGE from the FANTOM Consortium across 411 different tissues and cellular contexts, which by definition exclude regions near known transcription start sites and exons of mRNAs (both protein-coding and noncoding) and lncRNAs [13]. We subdivided their 38,538 robust phase 1 enhancers based on the number of contexts in which each was found to be active. We defined the top 5% most active enhancers as the “broadly active” set; this corresponded to 1961 enhancers with activity in greater than 45 contexts. As 15% of enhancers had activity in a single context, we randomly picked 1961 of these to be the “context-specific” or “narrow” enhancers.

We generated several sets of random non-enhancer regions for each enhancer set, using *shuffleBed* [25] to obtain length-matched regions for each input set of genomic regions. We also generated negative regions matched on GC content, chromosome distribution, and length using a custom script. We excluded all locations in the positive set as well as all enhancers from the full permissive CAGE enhancer dataset (43,011 total sequences), ENCODE blacklist regions, genome (hg19) assembly gaps, and experimentally verified VISTA enhancers (downloaded in March 2014) [26] from the negatives. Further, classifiers trained excluding known transcription start sites and exons [27] from the negatives were also able to accurately distinguish broadly active (Additional file 1: Figure S1), and there was a strong correlation between the weights assigned to each 6-mer between the two classifiers (Spearman’s ρ = 0.91, *P* < 2.2E–308), suggesting that they learned similar models of sequence.

To enable comparison with the fold enrichment analyses carried out by Yáñez-Cuna et al. (2014), we analyzed two additional human enhancer sets. We obtained DNase I hypersensitivity peaks and enhancer-associated histone modification data [15] from ENCODE (https://genome.ucsc.edu/ENCODE). Using *intersectBed* [25], we defined 13,069 broadly active DHS peaks found in at least 120 cell types, and 1449 regions containing both H3K27ac and H3K4me1 marks that were active in at least 10 cell lines: GM12878, H1hesc, Hmec, Hsmm, Huvec, K562, Nha, Nhlf, Nhek, and Osteoblast. We also filtered both sets to exclude regions overlapping CpG islands from the CpG Islands track in the UCSC Genome Browser. Many of the DHS peaks are expected to be enhancers, but this set includes other regulatory regions as well. We generated matched negative regions for these sets using the criteria described above for CAGE enhancers.

### DRM definition and identification

We searched for DRMs using position weight matrices (PWMs) with probability of one for the appropriate nucleotide in each position: CACACA, GAGAGA, GCGCGC, and TATATA. We identified and counted DRM occurrences using the python package MOODS, which searches input DNA sequences on both strands for occurrences of motifs defined by PWMs [28, 29], with a pseudocount of 0.001 and a match cutoff of *P* < 1/1024. Considering both strands meant that instances of the GC and TA repeats were counted twice, as they are their own reverse complements. We used the human genomic nucleotide frequencies for the background probabilities when calculating match scores and *P*-values, since the human genome is 42% GC.

We settled on these parameters after evaluating different combinations of thresholds and background frequencies with respect to the number and sequence diversity of DRMs we found. Using *P* < 1/4096 resulted in no perfect matches to the TA DRM passing the significance threshold, due to the higher genomic background frequency of TA bases (Additional file 1: Figure S2). Thus, we chose *P* < 1/1024 as it minimized the number of inexact matches included, while still allowing all perfect matches to pass the cutoff. Results were similar when identifying only exact matches (Additional file 1: Figure S3). We explicitly controlled for length in most analyses, because length is positively associated with activity in the FANTOM dataset. This step was unnecessary for relative fold enrichment analyses, as we compared relative occurrences in length-matched positive and negative sets.

Our parameters for defining DRMs differ from those used in Yáñez-Cuna et al. (2014), where they assumed an equal background probability for each nucleotide and used a PWM match cutoff of *P* < 1/256 [16, 30]. In addition, we used an invariant CA repeat motif, rather than the more variable motif inferred from STARR-seq data (Additional file 1: Figure S2). We believe that considering the background human genome nucleotide frequencies is necessary, due to the non-uniform GC content genome-wide and in enhancers. We also chose to use a stricter threshold (*P* < 1/1024) for identifying matches to DRM motifs, because lower thresholds, such as 1/256, allowed many diverse, non-repetitive motifs to match. This is a partial cause of the lower DRM density we observed compared to Yáñez-Cuna et al. (2014). Additionally, using invariant motifs of consistent length and information content for all four DRMs facilitated direct comparison of the results for different DRMs. We felt that these settings best captured the notion of a “dinucleotide repeat motif.” Other than these differences, the parameters used were the same as in Yáñez-Cuna et al. as best as we could determine.

### Fold enrichment analyses

We calculated motif fold enrichment (FE) by dividing the mean count of the occurrence of the sequence in question for the enhancer set by that in the negative set, which was either the matched non-enhancer regions from the genomic background or the context-specific enhancers. When we were comparing enhancers to genomic backgrounds, we analyzed four independent negative sets separately, and then plotted the mean and standard deviation of the log_2_(FE). For consistency with the enhancer prediction analyses, we analyzed 600 bp regions centered on each enhancer for all enhancer sets. In the broad vs. context specific comparison, all context-specific enhancers (activity = 1) were used. *P*-values were calculated for the distribution of counts in broadly active enhancers vs. a negative set by the Wilcoxon rank sum test. All violin plots are scaled by area.

### Enhancer prediction

To predict whether occurrence patterns of short DNA sequence motifs were sufficient to distinguish broadly active enhancers from the genomic background and from context-specific enhancers, we trained 6-mer spectrum kernel SVMs [31]. The spectrum kernel is a string kernel that defines the similarity of two DNA sequences based on the occurrence of all possible short DNA sequence patterns of a given length, *k,* within them. We computed the weight given to each possible 6-mer by each SVM [32] and averaged the weights across training runs vs. four independent negative sets. For predictions using DRMs, we used the counts per base pair for each DRM as training features. To predict enhancers based on counts of motifs of known transcription factors, we used 2911 PWMs from three databases: JASPAR 2016 vertebrate database [33], CIS-BP [34], and ENCODE [35]. We counted the occurrences of each motif in our genomic regions of interest using FIMO under default settings [36]. We then used the motif counts per base pair as features for the classifier.

Performance of all SVM classifiers was evaluated using 10-fold cross-validation, which limits overfitting by only training the classifier on a subset of the data at any given time. Receiver Operator Characteristic (ROC) and Precision Recall (PR) curves were calculated by averaging over the 10 cross-validation runs. All SVM analyses were performed using the SHOGUN Machine Learning Toolbox v4.0.0 [37]. For the predictions of broadly active regions versus context-specific regions, we took a random subset of the larger set to maintain the number of regulatory regions considered across analyses. We controlled for length differences by expanding or contracting enhancers in each set to be 600 bp long while maintaining their original centers; this was necessary due to a positive correlation between enhancer length and activity (Additional file 1: Figure S4).

### Transcription factor binding motif and expression analysis

We obtained transcription factor binding motif PWMs from the JASPAR 2016 vertebrate database [33], CIS-BP [34], and ENCODE [35]. In total, we considered 2911 motifs representing 1463 TFs. We obtained tissue specificity scores (TSPS) for 1326 TFs from the FANTOM Consortium [38]. A TF with uniform expression across all tissues was assigned a TSPS equal to zero, while a TF expressed in only a single tissue received a maximum TSPS of ~5. We classified TFs as “specific” (TSPS ≥1) or “broad” (TSPS <1) in their expression (Additional file 1: Figure S5), following the threshold used in the original publication [38].

We matched motif sequences to TF expression data using a combination of computational name matching and, where that failed, hand curation. For all motif names that did not have a match in the expression data, we identified synonyms and alternate names from Ensembl (Release 88) [39] and searched the expression data for any matches. Any TSPS or motif that could not be conclusively paired after manual inspection was discarded. For cases where a motif represented complex of TFs that had individual TSPS values, we assigned the greater of the two values, on the assumption that a complex cannot have wider expression than its most specific component. Ultimately, we obtained 1837 motifs for 563 unique TFs or complexes with TSPS expression values, of which 313 were broad and 250 were specific. The full list of motifs and TSPS values is given in Additional file 2: Table S1. For TFs with multiple motifs, we considered the mean GC and CpG content over all motifs. We compared the mean GC and CpG content distributions of the specific and broadly expressed TF groups using the Wilcoxon rank sum test. To determine whether CpG contributed any additional information to a model using GC content to predict TF expression, we calculated the squared semipartial correlation for two models. The first used TSPS as a continuous variable to represent expression while the second used dummy-coding to categorize TFs as broad or specific.

## Results

### DRMs are enriched (GC) and depleted (TA) in human enhancers, but the patterns do not match those in *Drosophila*

Recent work in *Drosophila* suggested that DRMs are a general feature of enhancers and that presence of many DRMs in an enhancer is a main driver of broad regulatory activity across diverse tissues [16]. To test the hypothesis that high DRM occurrence drives broad enhancer activity across tissues in humans [16], we analyzed sequence patterns in putative enhancers across diverse human cells and tissues. We considered 38,538 transcribed enhancers identified via CAGE for 411 contexts by the FANTOM consortium [13]. We defined the 1961 enhancers in the top 5% of the breadth of activity distribution (active in more than 45 contexts) as broadly active.

As a first step in investigating the contribution of DRMs to human enhancer activity, we computed the relative enrichment of DRMs in broadly active enhancers compared to context-specific enhancers and length-matched background regions using position weight matrices (PWMs). *Drosophila* enhancers exhibit enrichment for all DRMs except TA, and also show a positive association between DRM frequency and breadth of activity most strongly with GA and CA repeats, and GC to a lesser extent. Thus, under the *Drosophila* DRM model, we would expect CA, GA, and GC DRMs to be enriched in broadly active human enhancers compared to the other sets.

**Fig. 1:**
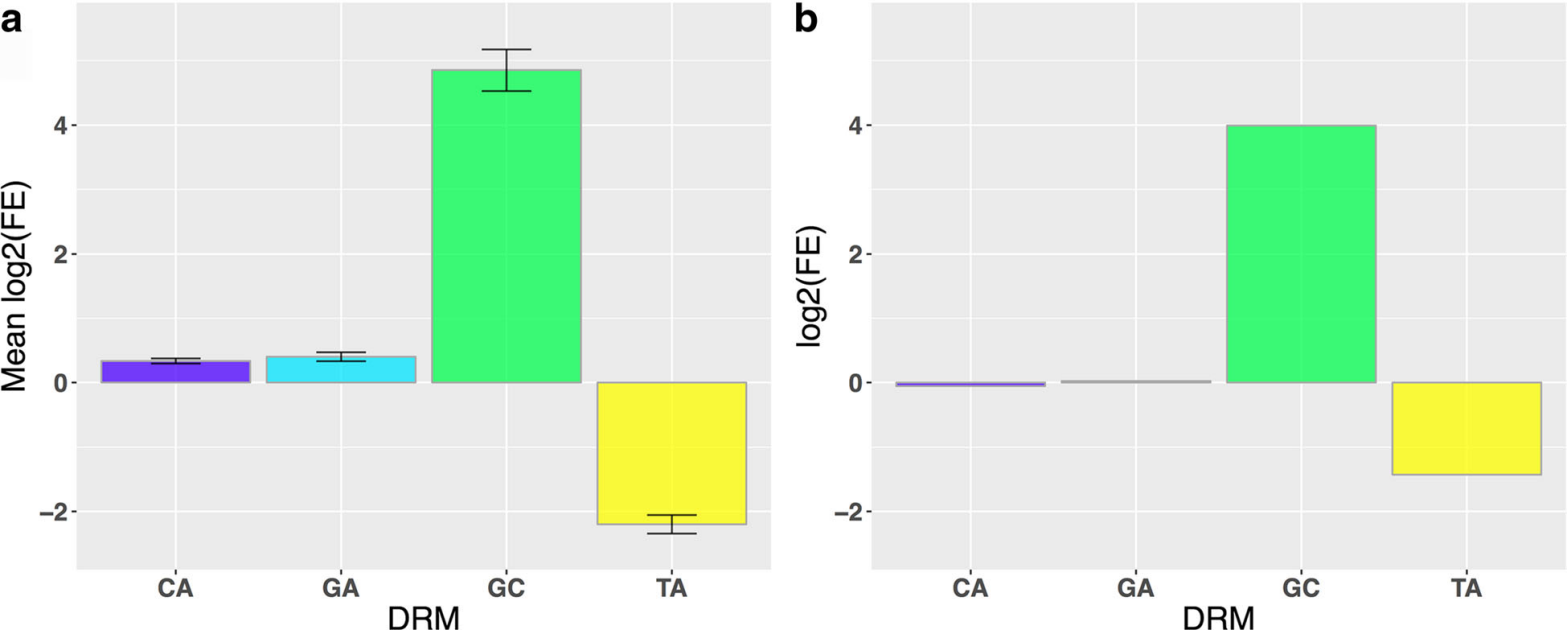
GC DRMs are enriched and TA DRMs depleted in broadly active enhancers. **a** The mean log_2_(Fold Enrichment) of the occurrence of each DRM in broadly active enhancers vs. genomic background. **b** The log_2_(Fold Enrichment) of the occurrence of each DRM in broadly active enhancers vs. context-specific enhancers. Error bars represent standard errors over four replicates

In humans, the CA, GA, and GC DRMs were all significantly enriched in broadly active enhancers compared to the genomic background (Fig. 1a; *P* = 2.0E–14, *P* = 2.3E–14, and *P* 1.7E–59, respectively). However, the magnitude of the enrichments for CA (1.3×) and GA (1.3×) were modest compared to GC (31.1×), and when compared to context-specific enhancers, significant enrichment remained only for the GC DRM (Fig. 1b, 15.9×, *P* = 1.1E–128). The TA DRM, on the other hand, was significantly depleted compared to both the genomic background (−4.5 x, *P* = 5.8E–34) and context-specific enhancers (−2.7×, *P* = 5.3E–35).

Furthermore, GC DRM density (DRM/bp) significantly increased as breadth of activity increased (Additional file 1: Figure S6; Spearman’s ρ = 0.12, *P* = 3.0E–114), indicating that this is a general trend across enhancers. Similarly, TA DRM density significantly decreased with breadth of activity (Spearman’s ρ = −0.020; *P* = 9.72E–05). There was not a significant trend for GA and CA DRMs (Additional file 1: Figure S6).

To confirm that the observed trends in DRM patterns were not unique to the transcribed enhancers defined by CAGE, we also analyzed DRM patterns in a “histone-derived set” of 1449 enhancers, consisting of regions with overlapping H3K4me1 and H3K27ac histone marks from 13 contexts [40], and a “DHS set,” of 13,069 DNaseI hypersensitive peaks across 126 contexts from ENCODE [15]. The DRM enrichment patterns were similar in these enhancer sets to those observed for CAGE enhancers: GC was significantly enriched and TA significantly depleted (Additional file 1: Figure S7).

The majority of the broadly active histone-mark-defined enhancers contained at least one DRM (Additional file 1: Figure S7); this is in contrast to their relative rarity in the CAGE and DHS sets. The increased counts are likely due to the greater length (and presumably lower resolution) of the histone-derived set: average enhancer length of 5797 bp vs. 200 and 297 bp for the DHS and CAGE sets, respectively. In contrast, the *Drosophila* enhancers were 500 bp long and had median DRM counts between 1 and 6, which is more similar to the histone set despite being an order of magnitude smaller [16, 41].

Overall, DRM patterns in human enhancers do not match the patterns observed for *Drosophila* enhancers, where CA, GA, and GC DRMs all showed enrichment in broadly active enhancers [16]. However, it is possible that DRMs in general maintain importance in driving broad enhancer activity between these diverse species, but the specific motifs are not conserved.

### DRMs alone are weakly predictive of broadly active human enhancers

**Fig. 2:**
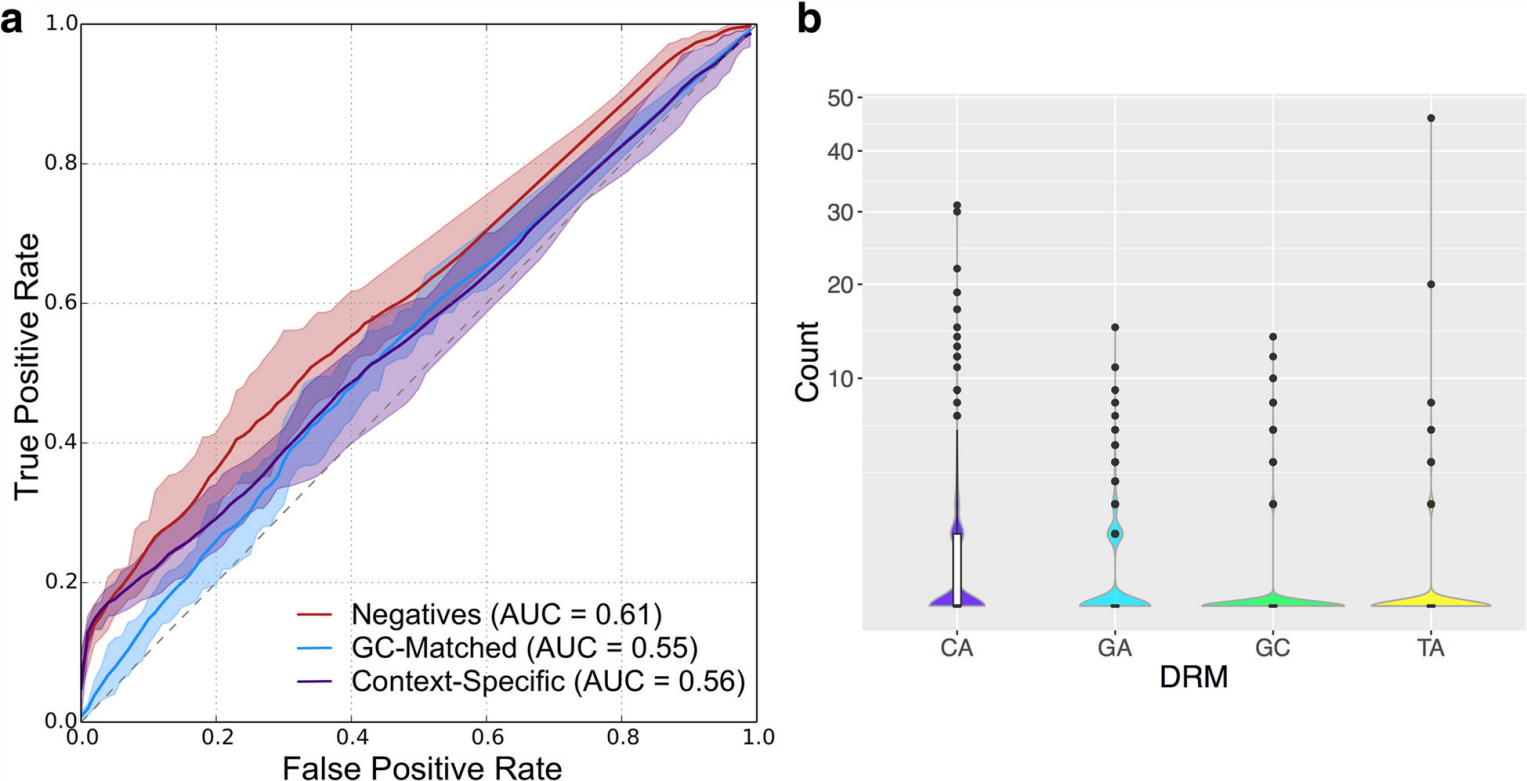
DRMs are not major drivers of broad enhancer activity in humans. **a** ROC curves for SVM-based classification of broadly active enhancers vs. length-matched genomic background (red), GC-matched genomic background (blue), and context-specific enhancers (purple) using the frequency of the four DRMs as features. The area under each curve (AUC) is given in parentheses. Shaded areas are bounded by the maximum and minimum observed ROC. Precision-recall curves are given in Additional file 1: Figure S8. **b** The distribution of occurrences for each DRM observed over 1961 broadly active enhancers. Most enhancers do not contain each class of DRM. Box plots show median and 1st/3rd quartiles

To directly evaluate the ability of DRMs to identify broadly active human enhancers, we used a support vector machine (SVM) learning framework [17]. We trained a linear SVM classifier to distinguish broadly active enhancers from context-specific enhancers and the genomic background using patterns of DRM occurrence. Using only DRM counts as features yielded poor performance at each classification task (Fig. 2a and Additional file 1: Figure S8A). We first trained the SVM to distinguish broadly active enhancers from a set of length-matched genomic background regions that excluded all putative enhancers, gaps in the genome assembly, and ENCODE blacklist regions (Methods). In 10-fold cross validation, the classifier performed poorly; it achieved an area under the receiver operating characteristic curve (ROC AUC) of 0.61 and a precision recall (PR) AUC of 0.64.

Enhancers are known to have greater GC content compared to the genomic background (mean 46% vs. 42%), so to determine whether DRM sequence patterns held predictive value independent from overall GC content, we repeated the previous analysis training the classifier on negative training sets generated from random background regions matched to broadly active enhancers for both length and GC content. This classifier had decreased performance compared to the non-GC-matched classifier (Fig. 2a and Additional file 1: Figure S8A, *P* = 6.2E-8), suggesting that GC content was important for some for the predictive ability of DRMs.

Next, we evaluated the ability of DRMs to distinguish broadly active enhancers from context-specific enhancers. Since the context-specific enhancers were shorter on average (Additional file 1: Figure S4), we controlled for length by expanding or contracting all enhancers in both sets to be 600 bp long, approximately the mean length of the most active enhancers. This DRM-based classifier trained vs. context-specific enhancers also performed poorly: ROC AUC of 0.56 and PR AUC of 0.61 (Fig. 2a and Additional file 1: Figure S8A). Because DRMs were rare in broadly active enhancers (median occurrence of zero for all DRMs; Fig. 2b), the poor performance of the DRM-based SVM is not surprising, and it suggests that DRMs are not major drivers of enhancer activity in humans.

### Comprehensive analysis of short DNA sequence motif occurrence accurately identifies broadly active human enhancers

**Fig. 3:**
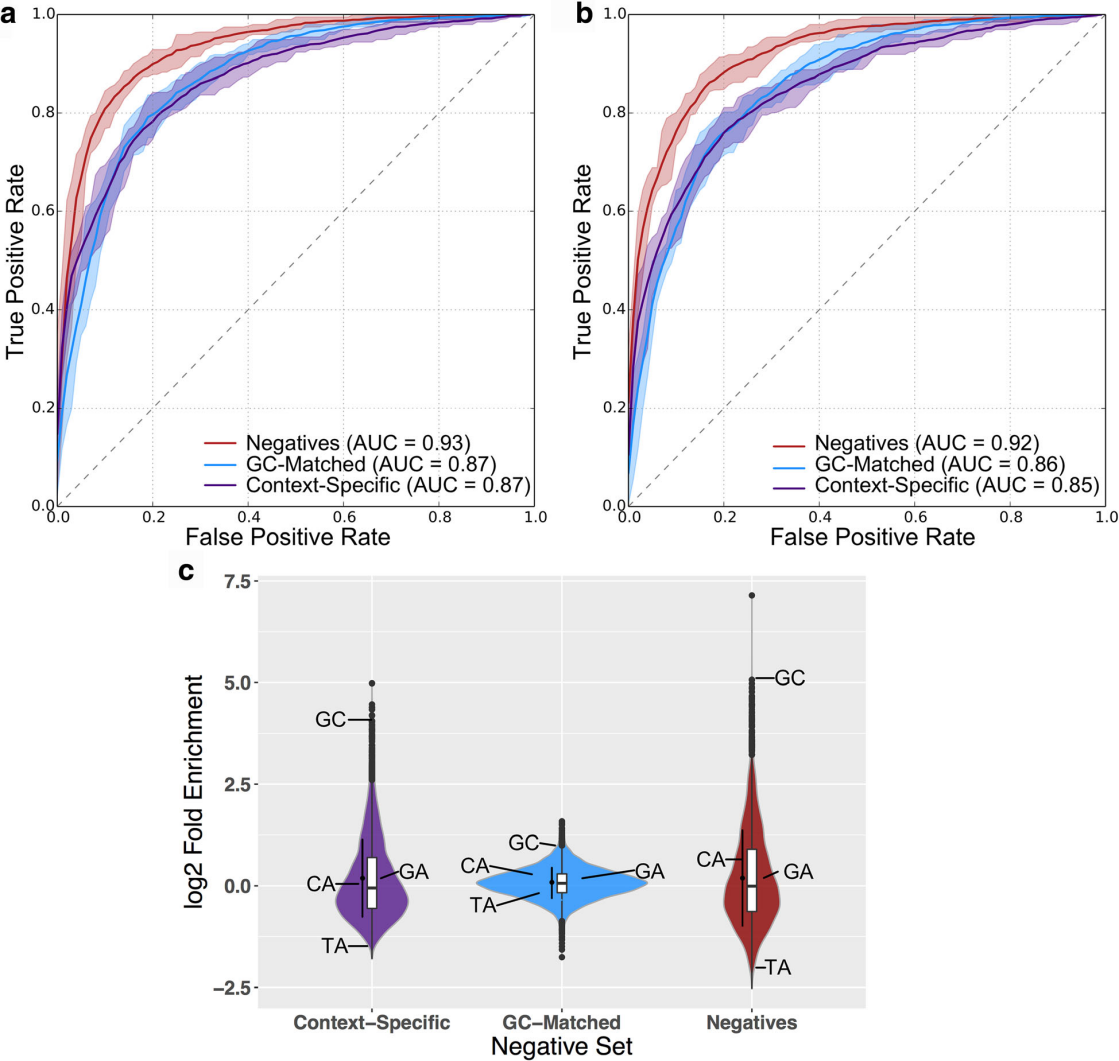
Short DNA sequence patterns accurately distinguish broadly active human enhancers from the genomic background and context-specific enhancers. Classifiers trained using (**a**) all possible 6-mers or (**b**) the density of TF motifs as features can identify the broadly active human enhancers. ROC curves were calculated using 10-fold cross-validation and averaging the ROC obtained by each round of validation. The area under each curve (AUC) is given in parentheses. Shaded areas are bounded by the maximum and minimum observed ROC. Precision-recall curves are given in Additional file 1: Figure S8. (**c**) The log_2_(Fold Enrichment) of all 6-mers in the 1961 broadly active enhancers vs. the corresponding negative sets. Enrichments were calculated for each of the 4096 6-mers. Box plots show median and 1st/3rd quartiles, while the black point and line indicate mean and standard deviation. The fold changes for the four DRMs are indicated on each distribution by GC, CA, GA, and TA

Given that DRMs by themselves were only weakly predictive of broadly active human enhancers, we evaluated the ability of additional short DNA sequence motifs to predict the breadth of enhancer activity. Using the occurrence patterns of all 4096 possible 6-mers in the enhancer sequence as features in a spectrum kernel SVM [31], we repeated the classifications performed for the DRMs. The classifier trained on broadly active enhancers vs. random background regions performed very well (Fig. 3a and Additional file 1: Figure S8B; ROC AUC = 0.93, PR AUC = 0.92). When classifying GC-matched regions, the performance of this classifier decreased, but was still strong (Fig. 3a and Additional file 1: Figure S8B; ROC AUC = 0.87, PR AUC = 0.86, *P* = 8.3 E-15). Furthermore, the classifier performed as well at distinguishing between broadly active and context-specific enhancer classes as it did distinguishing broadly active enhancers from GC-matched genomic background (Fig. 3a and Additional file 1: Figure S8B; ROC AUC = 0.87, PR AUC = 0.88, *P* = 0.61).

To test whether considering all possible 6-mers increased performance compared to using current knowledge of TF binding preferences, we evaluated the performance of classifiers trained using counts of matches to 2911 known TF binding motifs (Methods). All three classifiers performed very slightly, but significantly, worse in these analyses than when considering all 6-mers (Fig. 3a vs. 3b; negatives *P* = 0.0245, GC-matched *P* = 7.5E–3, context-specific *P* = 3.2E–5). Thus, limiting the training features to current knowledge of TF specificity only modestly decreased performance.

Since the relative performance of the classifiers indicates that DRMs are not the strongest contributors to enhancer sequence activity patterns, we evaluated their contribution to human enhancer activity in the context of all possible 6-mers (Fig. 3c). The enrichment of the GC DRM in broadly active enhancers was more than two standard deviations (SDs) above the mean over all 6-mer enrichments for all three comparisons. The TA DRM was more than 1 SD less than the mean for the broadly active enhancers vs. genomic background and context-specific enhancers (Fig. 3c). The CA and GA DRMs were both within 1 SD of the mean for all three comparisons. This suggests that the GC DRM, and to a lesser extent the TA DRM are enriched and depleted, respectively, in broadly enhancers compared to the enrichment of 6-mers in general. Despite this enrichment, the rarity of DRMs in broadly active enhancers (Fig. 2b) reduced their predictive ability overall. Collectively, these results show that DRMs alone are not nearly as informative about enhancer activity and breadth as models that include additional short sequence patterns or known TF binding motifs.

### GC-rich motifs are predictive of broadly active enhancers

**Fig. 4:**
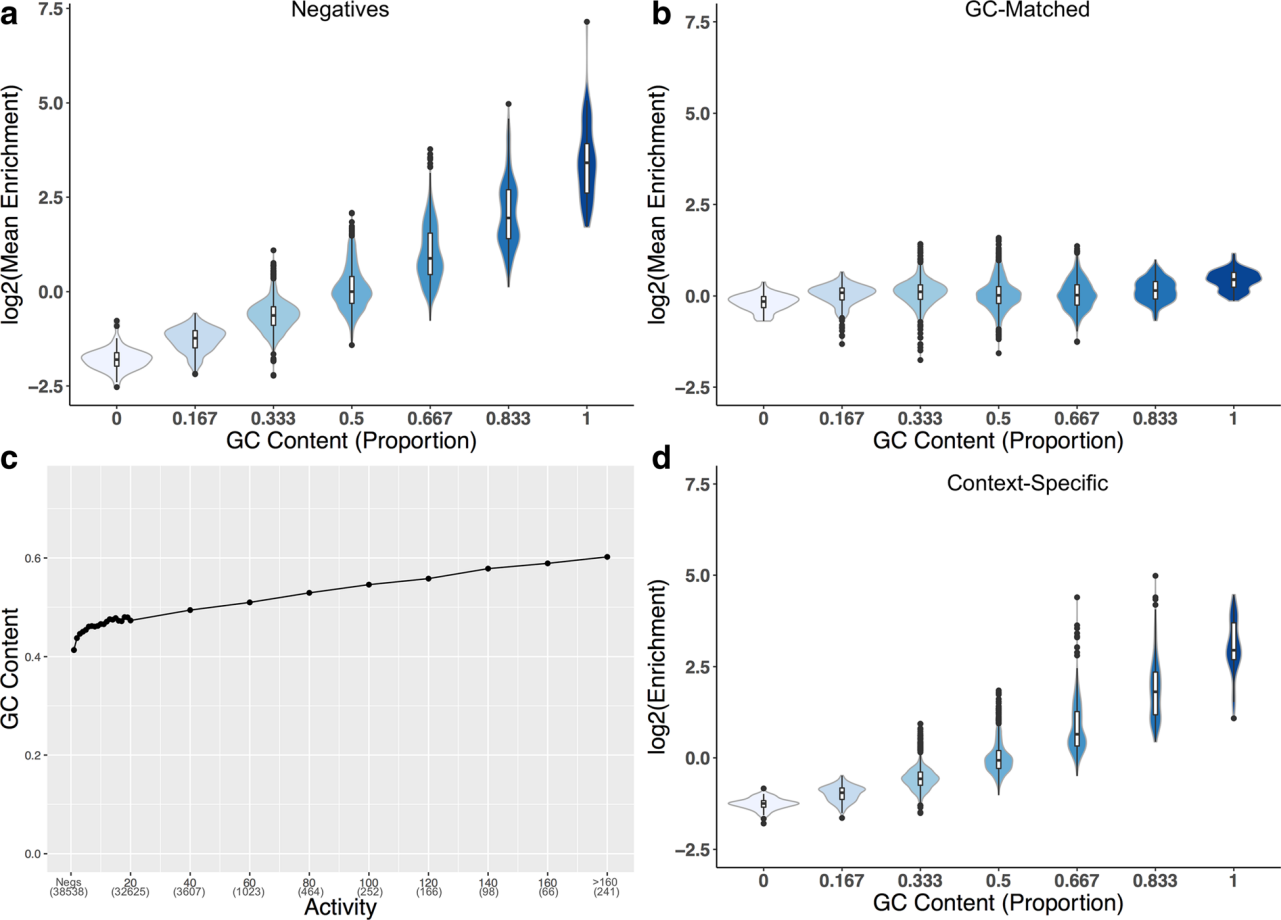
Short DNA sequence patterns enriched in broadly active enhancers have high GC content. **a** The log_2_ of the fold enrichment for each 6-mer (*N* = 4096) is plotted against its GC-content for comparison of broadly active vs. non-GC-matched background. **b** The same as (**a**) but comparing broadly active enhancers to GC-matched background regions. **c** Mean enhancer GC content is significantly correlated with breadth of activity among enhancers (Spearman’s ρ = 0.25, *P* < 2.2E–308). Enhancers were assigned to bins based on the number of contexts in which they were active (x-axis); each bin is labeled with the highest activity present in that bin, and the number of regions in it. **d** Same as (**a**), but comparing broadly active to context-specific enhancers. The number of 6-mers in each GC content bin is: 64 for 0 and 1, 384 for 0.167 and 0.833, 960 for 0.333 and 0.666, and 1280 for 0.5

Given the elevated GC content of enhancers and the enrichment and depletion of the GC and TA DRMs (the two DRMs with unequal GC content), we quantified the relationship between GC content and 6-mer enrichment in broadly active enhancers. In comparisons with the genomic background, the correlation was significantly positive (Fig. 4a; Spearman’s ρ = 0.87, *P* < 2.2E–308). This is not surprising given that enhancers have high GC content compared to the genomic background. As expected, this trend was strongly attenuated in the GC-matched comparison (Fig. 4b; Spearman’s ρ = 0.045, *P* = 0.004).

We previously observed that enhancer GC content varied in different tissues’ enhancers [17], and here we found that GC content is positively correlated with breadth of activity among the enhancers (Fig. 4c; Spearman’s ρ = 0.25, *P* < 2.2E–308). Similarly, GC content showed a high correlation with enrichment in broadly active vs. context-specific enhancers (Fig. 4d; Spearman’s ρ = 0.88, *P* < 2.2E–308). This mirrors the patterns shown by the GC and TA DRMs.

The classification function learned by a trained spectrum kernel SVM implicitly assigns weights to each 6-mer that indicate its contribution to the classifier’s prediction. Repeating the GC content analyses using these 6-mer weights rather than their enrichment resulted in similar correlations (Additional file 1: Figure S9; Spearman’s ρ = 0.31, 0.014, 0.29, *P* = 1.4E–93, *P* = 0.36, *P* = 4.5E–80 for genomic background, GC-matched, and context-specific enhancers respectively). This argues that, in terms of both individual motif enrichment and importance to trained classifiers, high GC content is characteristic of broadly active enhancers, regardless of status as a DRM.

### Broadly active TFs have GC-rich motifs

**Fig. 5:**
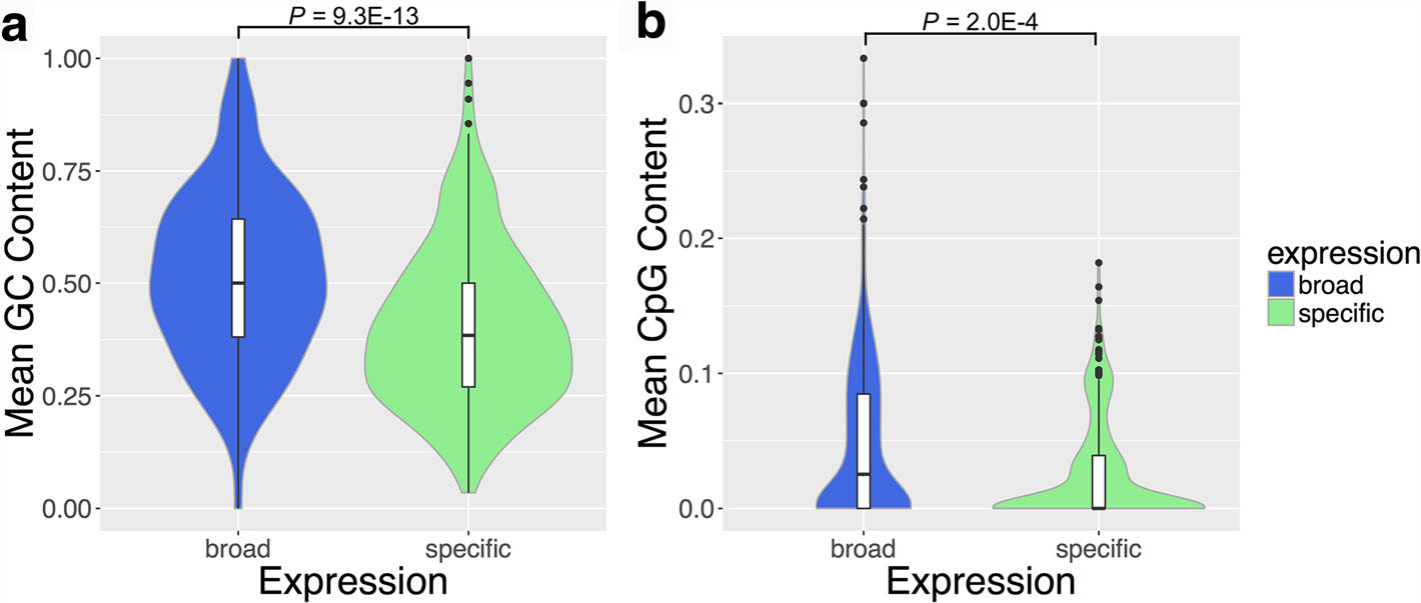
Binding motifs for broadly expressed TFs are more GC- and CpG-rich than motifs for context-specific TFs. The mean (**a**) GC content and (**b**) CpG content of motifs recognized by 313 broadly expressed TFs (blue) was significantly greater than that of 250 context-specific TFs (green). For TFs with multiple motifs, we took the mean over motifs. Box plots show median, and 1st/3rd quartile. *P*-values were calculated using the Wilcoxon Rank Sum test

The highly weighted/enriched motifs likely serve important biological functions that contribute to enhancer activity. Since enhancers function by binding transcription factors, we hypothesized that DNA sequence patterns that facilitate the binding of broadly expressed transcription factors could drive broad enhancer activity across many contexts. To explore this, we analyzed the sequences and breadth of expression of known TF binding motifs from three motif databases: JASPAR [33], CIS-BP [34], and ENCODE [35]. We classified the TFs into broadly expressed and tissue-specific classes based on expression data from the FANTOM Consortium [38]. In support of our hypothesis, the motifs of broadly active TFs have significantly higher GC content than those of context-specific TFs (Fig. 5a; 51% vs. 40%, *P* = 9.3E–13), mirroring the trend seen in 6-mers predictive of broad enhancer activity. The broad TF motifs also had higher CpG content than context-specific TF motifs (Fig. 5b; 0.05 vs. 0.03 per bp, *P* = 2.0E–4). This suggests that DNA methylation of those sites could play a role in regulating binding of broadly-active TFs [42]. However, a semipartial correlation analysis revealed that CpG content did not explain additional information about breadth of expression beyond what was expected from GC content (TSPS score *P* = 0.11, Broad vs. Specific *P* = 0.17).

## Discussion

We analyzed the contribution of DRMs and other short DNA sequence motifs to the activity patterns of human enhancers across hundreds of cellular contexts. In contrast to the model proposed in *Drosophila* [16], GC DRMs were enriched in broadly active enhancers compared to both the genomic background and context-specific enhancers, while TA DRMs were depleted. Using an unbiased machine learning framework, we found that DRM occurrence patterns were only weakly predictive of broadly active human enhancers (ROC AUC ranging from 0.55 to 0.61). However, a classifier trained on the occurrence of all possible 6-bp sequences very accurately distinguished broadly active human enhancers from the genomic background (ROC AUC = 0.93), GC-matched background regions (ROC AUC = 0.87), and context-specific enhancers (ROC AUC = 0.87). Furthermore, 6-mers highly predictive of broad activity tended to be GC-rich, while those with the most negative weights tended to be GC-poor, even when classifying GC-matched regions. These results suggest that broadly active enhancers have distinct sequence properties, and that the enrichment and depletion of DRM sequences is part of a larger pattern in which particularly GC-rich and GC-poor sequences are indicative of broad and context-specific activity, respectively. Consistent with this pattern, TFs with broad expression have greater affinity for GC- and CpG-rich motifs than TFs with tissue-specific expression patterns.

Our findings in human enhancers differ from recent results in *Drosophila* in several respects. Broadly active *Drosophila* enhancers exhibit enrichment for all DRMs except TA, while broadly active human enhancers are consistently enriched only for the GC DRM. We also found that DRM counts alone are significantly less predictive of enhancer activity than wider sequence patterns or *Drosophila* models including many motifs [16]. Other sequences predictive of broad enhancer activity tend to be GC-rich, demonstrating that the effects on human enhancer sequence activity are not unique to repeat elements.

There are several possible causes of the observed differences in DRM patterns between humans and *Drosophila*. First, they could be due to differences in the enhancer identification strategy used. The main set of human enhancers analyzed was identified using CAGE to detect native eRNAs, while the *Drosophila* enhancer sets were assembled using STARR-seq [41]. Both methods have potential weaknesses. CAGE-seq is only able to identify enhancers that produce bidirectional capped transcripts, while the STARR-seq assay isolates potential regulatory sequences in reporter constructs separate from their genomic contexts and thus could introduce activity patterns not representative of enhancers in their natural chromatin context. To address this concern, we analyzed other human enhancer sets defined using functional genomics data (histone modifications and DNaseI hypersensitivity data). We found patterns consistent with the CAGE enhancers, so this suggests that our findings are robust among human enhancers. Second, differences in the number of biological contexts considered could influence the comparison. We considered enhancer activity across 411 human cellular contexts, while only three cell types were considered in the *Drosophila* study. These cells were from different lineages and developmental stages, but further work that considers more cellular contexts in *Drosophila* would be necessary for a more direct comparison. Finally, there were a number of technical differences in how DRMs were defined between the studies. For example, we used stricter *P*-value thresholds for calling DRMs and a background model tailored to the genome GC content rather than uniform frequencies. We felt that these definitions better reflected the concept of a “dinucleotide repeat motif” and enabled comparison between different motifs. Nonetheless, we found that this and other technical differences did not influence our conclusions (Additional file 1: Figures S1–3).

Thus, while technical factors may have contributed, the observed differences were likely also influenced by biological differences between the *Drosophila* and human genomes. For example, despite having similar GC content, the *Drosophila* genome is not as CpG-depleted as humans [43]. This could influence the roles and dynamics of CpG islands in enhancer activity between the species. In addition, while recent studies of transcriptional networks and TF binding preferences have revealed remarkable conservation of elements of metazoan gene regulation [44–46], there are differences in the TF complement and gene expression patterns between these two species. It is possible that the differences in DRM enrichments reflect a difference in the sets of TFs that bind broadly active enhancers in the two species, or that broadly expressed transcription factors in *Drosophila* do not show the same collectively higher GC content compared to context-specific TFs (Fig. 5).

Since the motifs of broadly expressed human TFs have higher GC and CpG content than context-specific TFs, DNA methylation could play a larger role in determining their binding and activity. This raises the possibility that broadly active enhancers and TFs in mammals have evolved to be GC- and CpG-rich, perhaps influenced by the repressive role of CpG methylation. As DNA methylation in *Drosophila* is much less pervasive [47], TFs in *Drosophila* would not have the same pressure. More generally, high GC content could also facilitate broad activity by influencing DNA shape near binding motifs [48]. The differences in the evolution and function of DRMs, other regulatory sequence motifs, and GC content between humans, flies, and other organisms must be explored further, but such studies will require comprehensive catalogs of enhancers active across many tissues in species.

## Conclusions

We demonstrate that while short DNA sequence patterns can accurately identify broadly active human enhancers, DRMs are not the main drivers of activity. This emphasizes the importance of DNA sequence patterns on enhancer biology and suggests several avenues for future research. Most importantly, more work is needed to understand the regulatory logic of enhancer sequences; we suspect that highly predictive sequence patterns could be mined to identify novel binding motifs and combinatorial interactions. Our results also reveal that we understand relatively little about how enhancer sequence and activity evolve, especially in the context of DNA methylation. Resolving the evolution and mechanistic functions of these enriched sequences will require further statistical and experimental analyses, but the approach presented here provides a framework in which to quantify and explore how DNA sequence influences gene regulatory activity.

## Additional files

Additional file 1: Supplementary figures S1-S9. (PDF 1932 kb)

Additional file 2: Supplementary table S1. (TXT 54 kb)
