# Supplementary material for: Short DNA sequence patterns accurately identify broadly active human enhancers
Source: BMC Genomics. 2017 Jul 17;18:536. doi: 10.1186/s12864-017-3934-9 (PMC5512948; doi:10.1186/s12864-017-3934-9)
Supplement: Supplementary file 1 — Supplementary figures S1-S9. (PDF 1932 kb) [file 12864_2017_3934_MOESM1_ESM.pdf]

Supplementary Figures

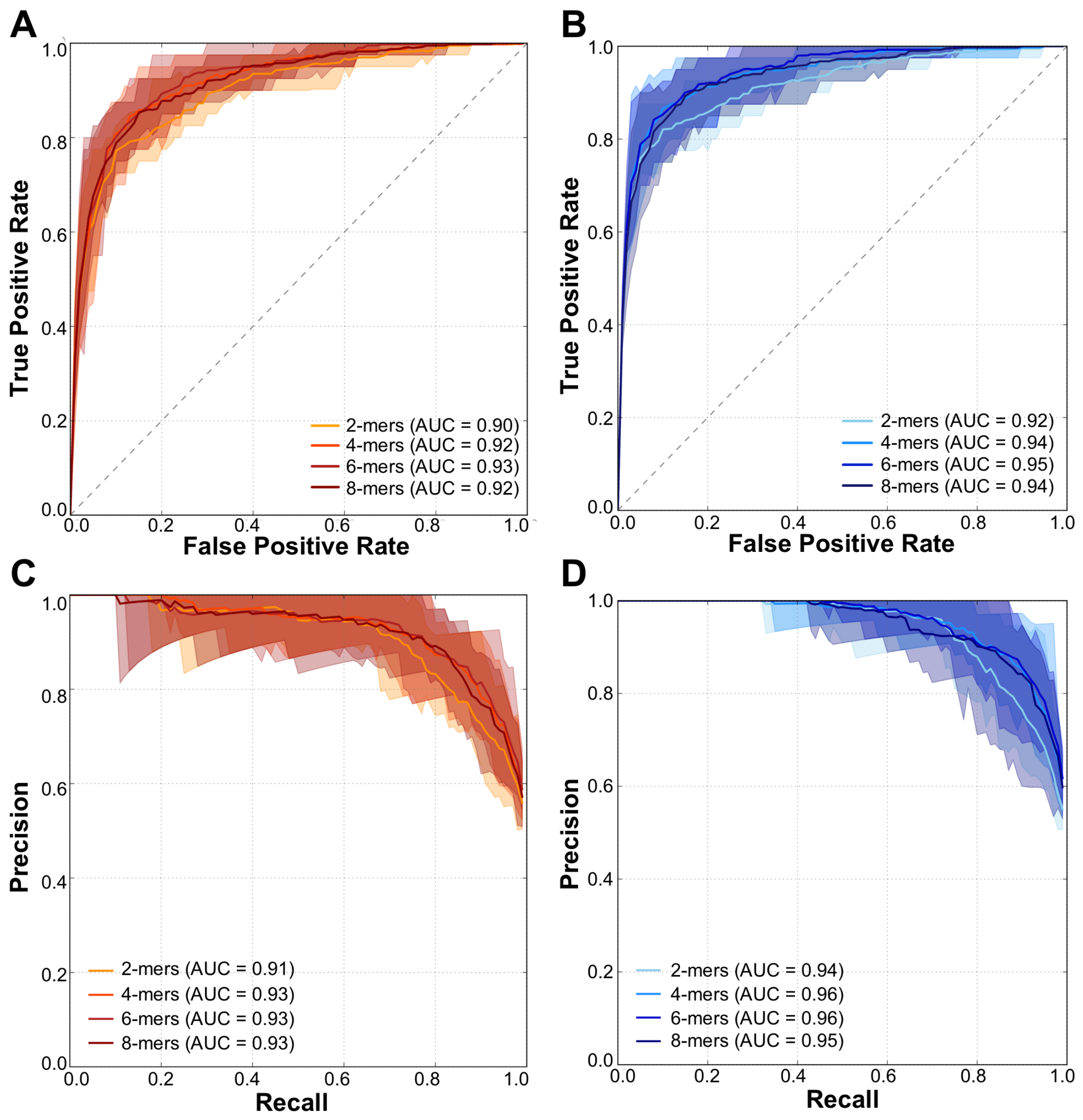

Figure S1. ROC Curves (A & B) and PR Curves (C & D) of classifiers trained using all possible 2-mers, 4-mers, 6-mers, or 8-mers as features. The positive training set for each was a set of 401 CAGE enhancers active in ≥120 cellular contexts, while the negative training set was a set of 401length-matched non-enhancer genomic regions generated excluding the full permissive CAGE dataset (43,011 total sequences), ENCODE blacklist regions, genome (hg19) assembly gaps, and experimentally verified VISTA enhancers (downloaded in March 2014) (A & C) and additionally Ensembl transcription start sites and exons (B & D). The solid curves represent the mean over 10-fold cross-validation. Shaded regions represent the minimum and maximum curves.

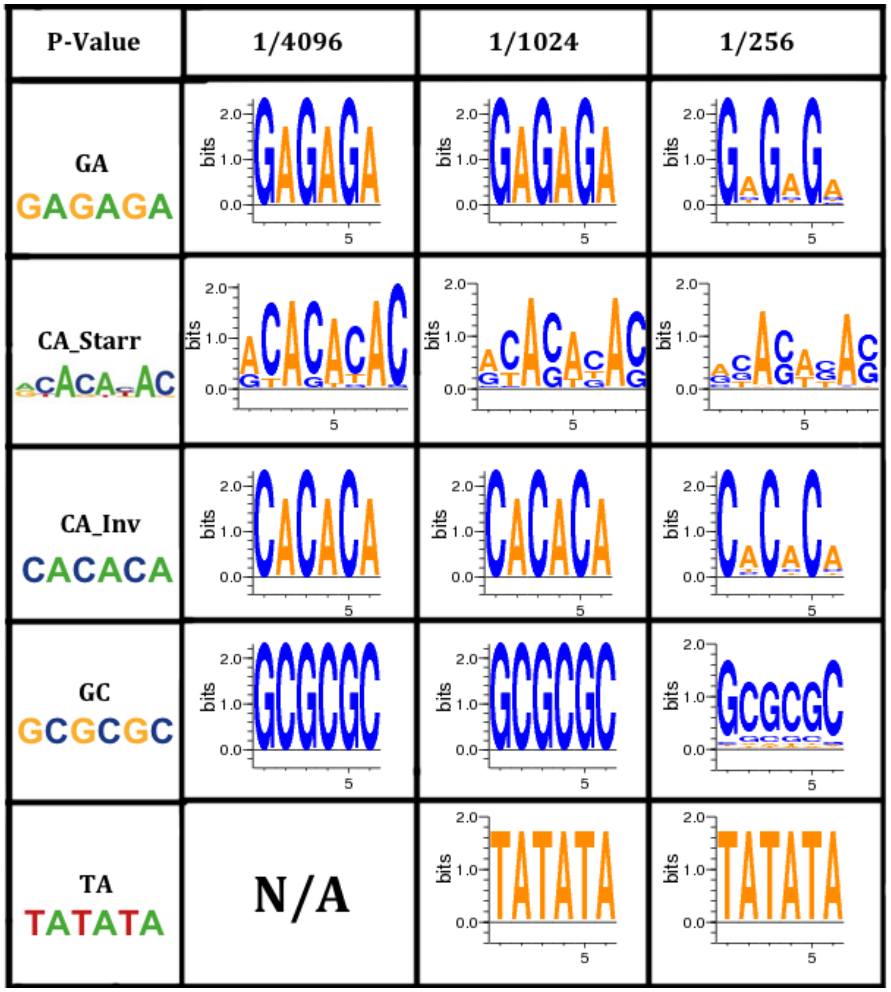

Figure S2. Sequence logos summarizing sequences matched to each motif (left column) at different threshold *P*-values (right columns) in 38,538 random genomic regions. We considered background human nucleotide frequencies in the *P*-value calculations (60% A/T, 40% C/G). N/A indicates that no matching sequences were found; this is due to the inability of even perfect matches to the TA motif to attain $P < 1/4096$ given the high background frequencies of A and T in the human genome. CA_Starr is the motif used for the CA DRM in Yáñez-Cuna et al. 2014, and CA_Inv is the form used here to be consistent with the definition of the other DRMs.

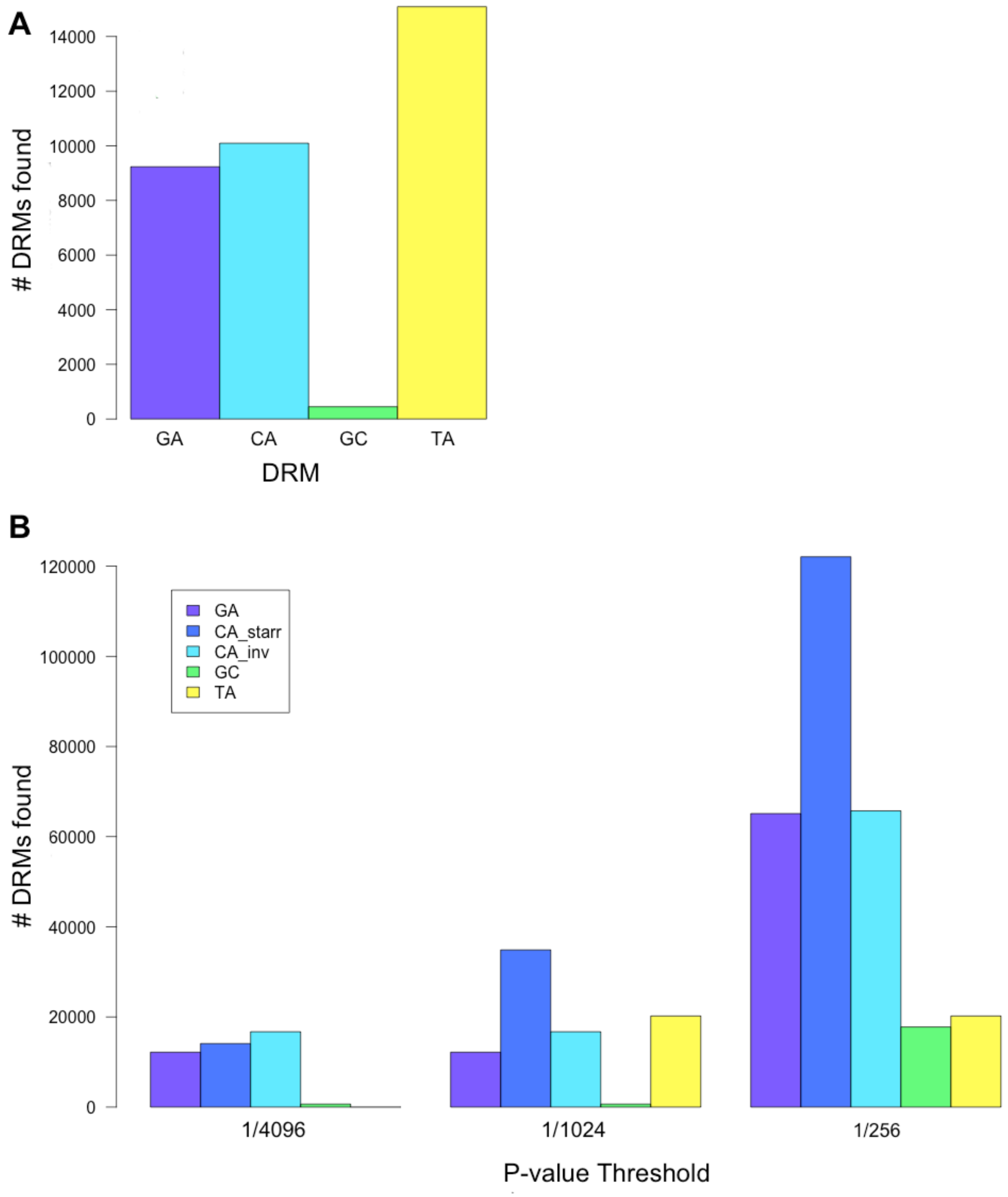

Figure S3. Methodology affects DRM counts. DRMs were identified in 38,538 random genomic regions using (A) regular expressions to find exact sequence matches, and (B) MOODS to find matches to the corresponding PWMs at different thresholds. For MOODS, we specified human background nucleotide frequencies. DRM matches were counted on both strands.

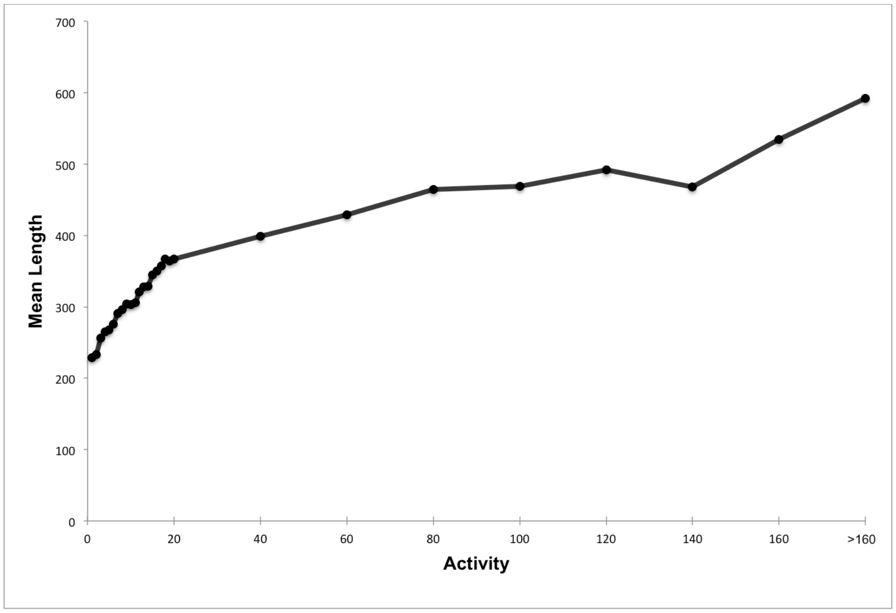

Figure S4. Mean enhancer length is significantly positively associated with activity in CAGE-defined enhancers (Spearman's ρ = 0.354, *P* < 2.2E-16). Enhancers were assigned to bins based on the number of contexts in which they were active (x-axis); each bin is labeled with the highest activity present in that bin.

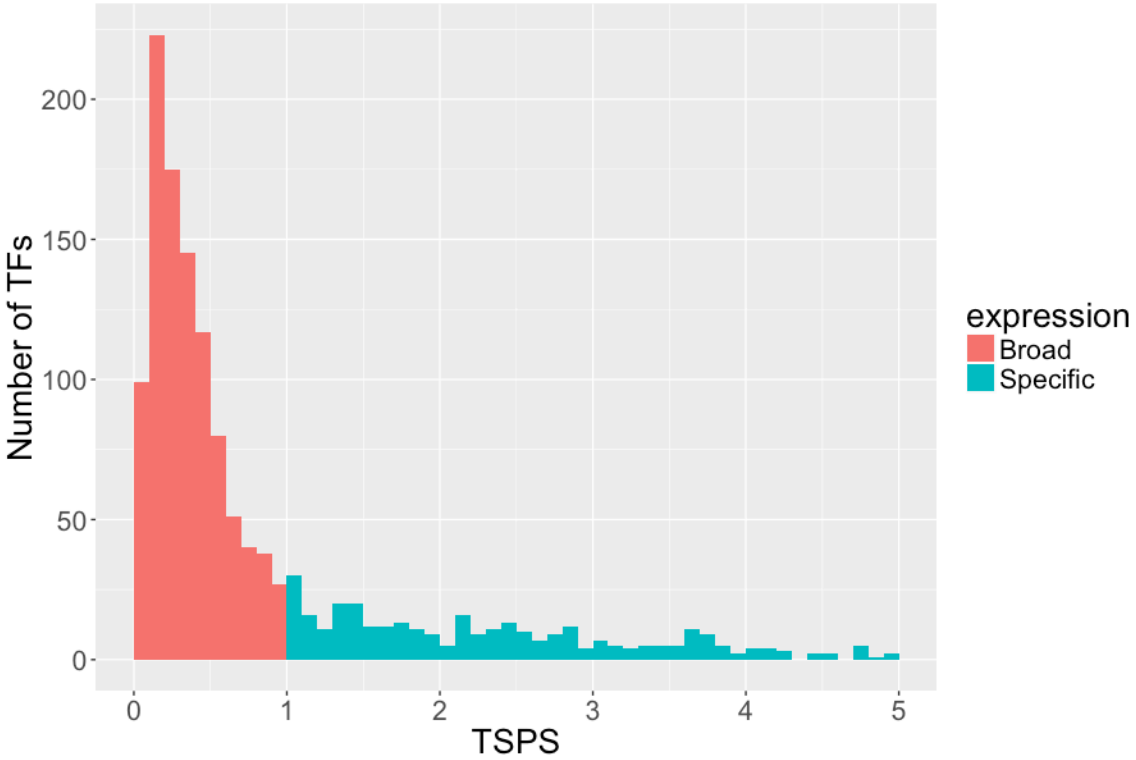

Figure S5. Distribution of TSPS scores in FANTOM TFs. TSPS ≥ 1 = 'specific' expression, TSPS < 1 = 'broad' expression.

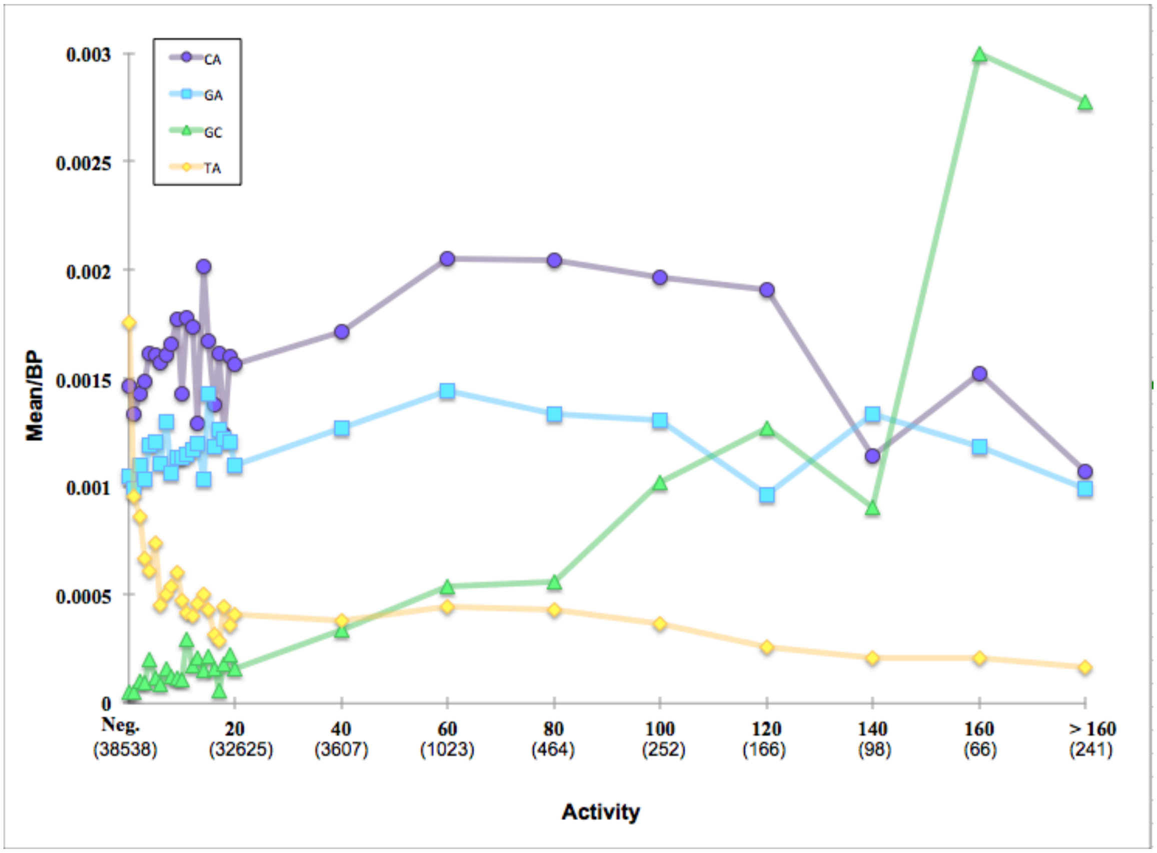

Figure S6. GC DRM density (green) significantly increases with increasing breadth of enhancer activity (Spearman's ρ = 0.12, *P* < 2.2E-16, while TA DRM density (yellow) significantly decreases (Spearman's ρ = –0.020; *P* = 9.72E-05). Enhancers were assigned to bins based on the number of contexts in which they were active (x-axis); each bin is labeled with the maximum number of tissues and number of enhancers (in parentheses) present in it. The values plotted at zero are from randomly generated non-enhancer (negative) regions. For enhancers active in ≤20 contexts, DRM densities for each activity level are plotted to provide additional resolution.

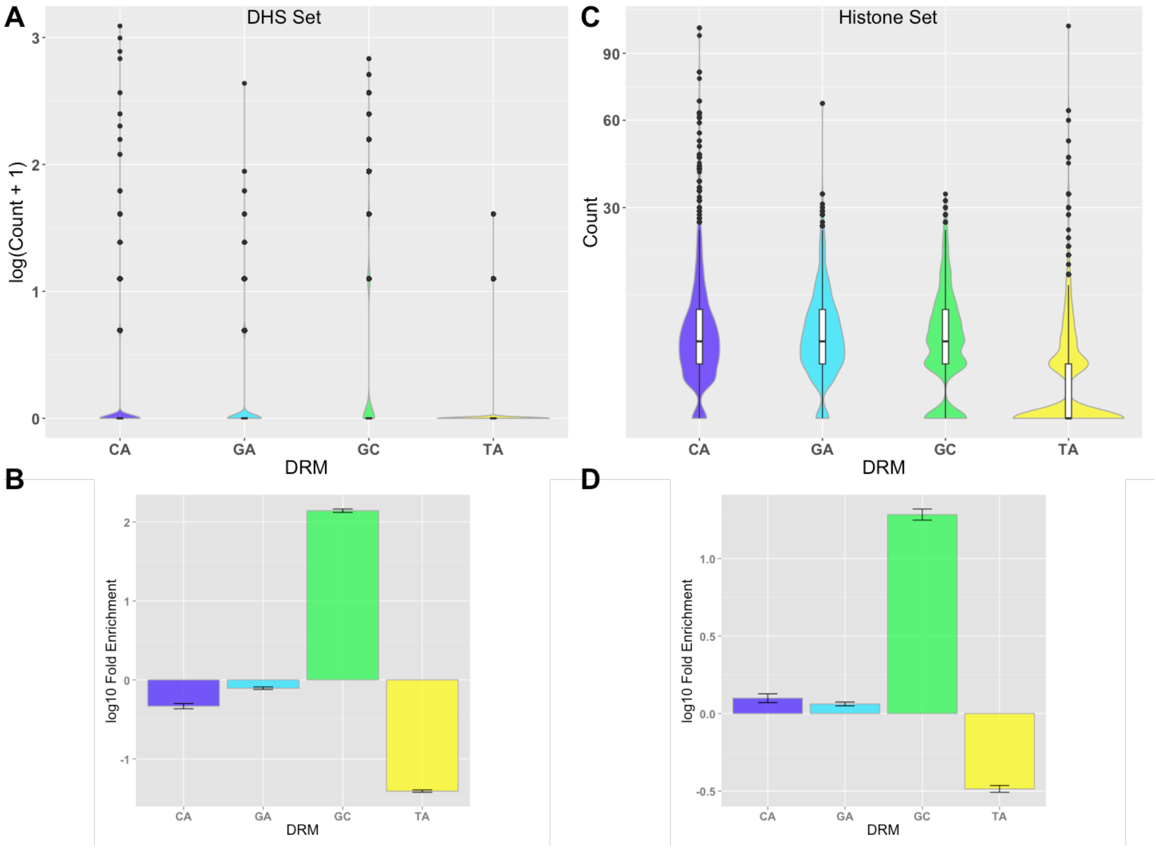

Figure S7. DRMs are significantly enriched (GC) and depleted (TA) in DHS and histone-defined regions in many cellular contexts. (A) The distribution of $\log_{10}$ of occurrences observed over all DHS peaks for each DRM. The majority of enhancers had a DRM count of 0 (78-99%), so we added a pseudocount of 1 to each for visualization purposes. (B) Log_{10}(fold enrichment) of the occurrence of each DRM in 13,069 broadly active DHS peaks compared to random length-matched non-enhancer regions. (C & D) Same as (A & B) but in 1449 overlapping H3K27ac and H3K4me1 peaks. Error bars show standard error of the mean over four replicates. For both sets, differences in CA, GC, and TA are significant ($P < 2.2\text{E-}16$, Wilcoxon Rank Sum)

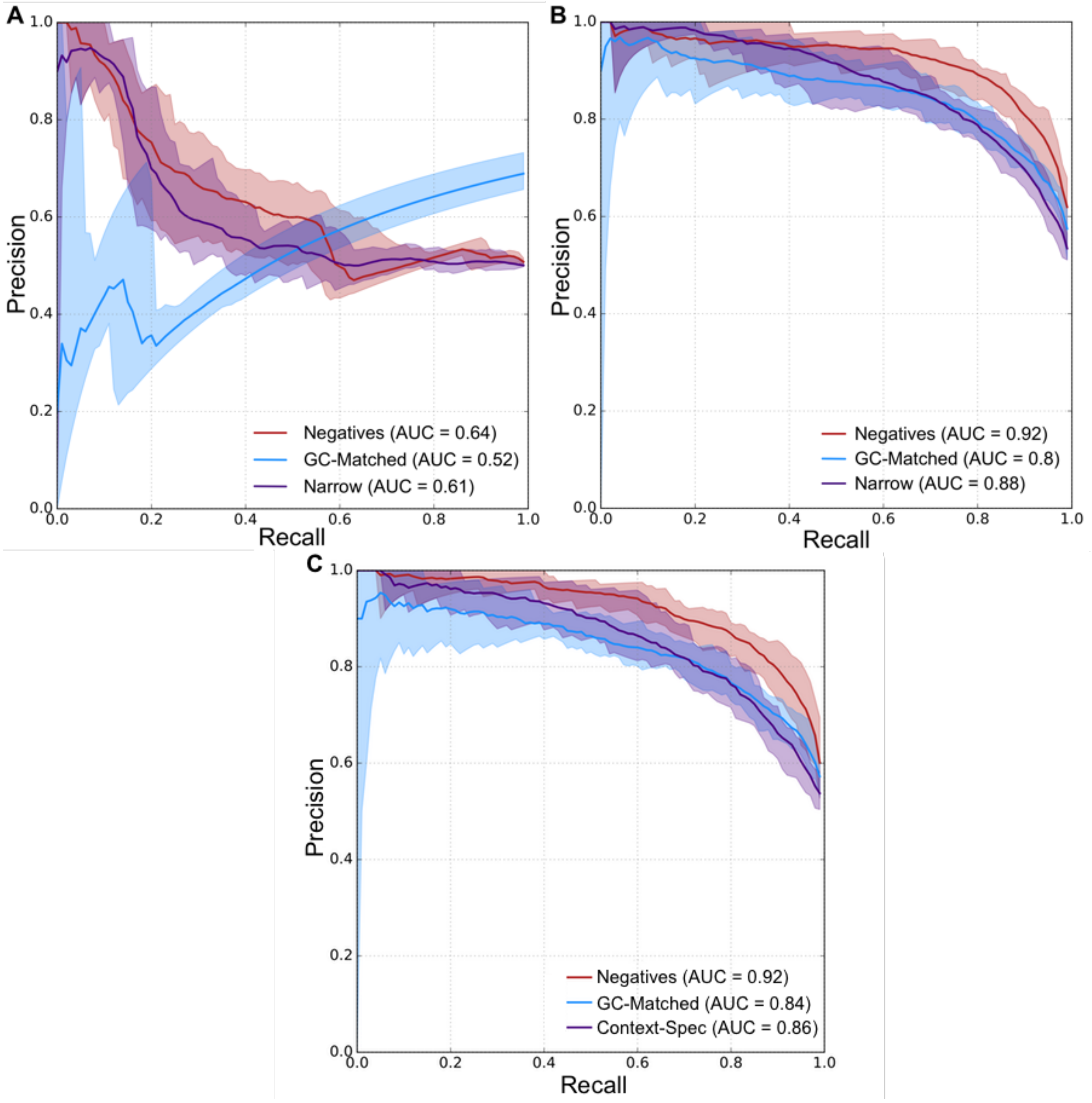

Figure S8. PR curves for SVM-based enhancer prediction in Figure 1 using A) density of DRMs, B) all possible 6-mers, or C) density of TF motifs as features, and trained with 401 broadly active CAGE enhancers as positives and 401 length-matched genomic background (red) or GC-matched genomic background (blue), or 401 context-specific enhancers (purple) as negatives. SVM classifiers based on the occurrence of DNA 6-mers in these regions were able to identify broadly active enhancers with significant accuracy. The solid curves represent the mean over 10-fold cross-validation. Shaded regions represent the minimum and maximum curves.

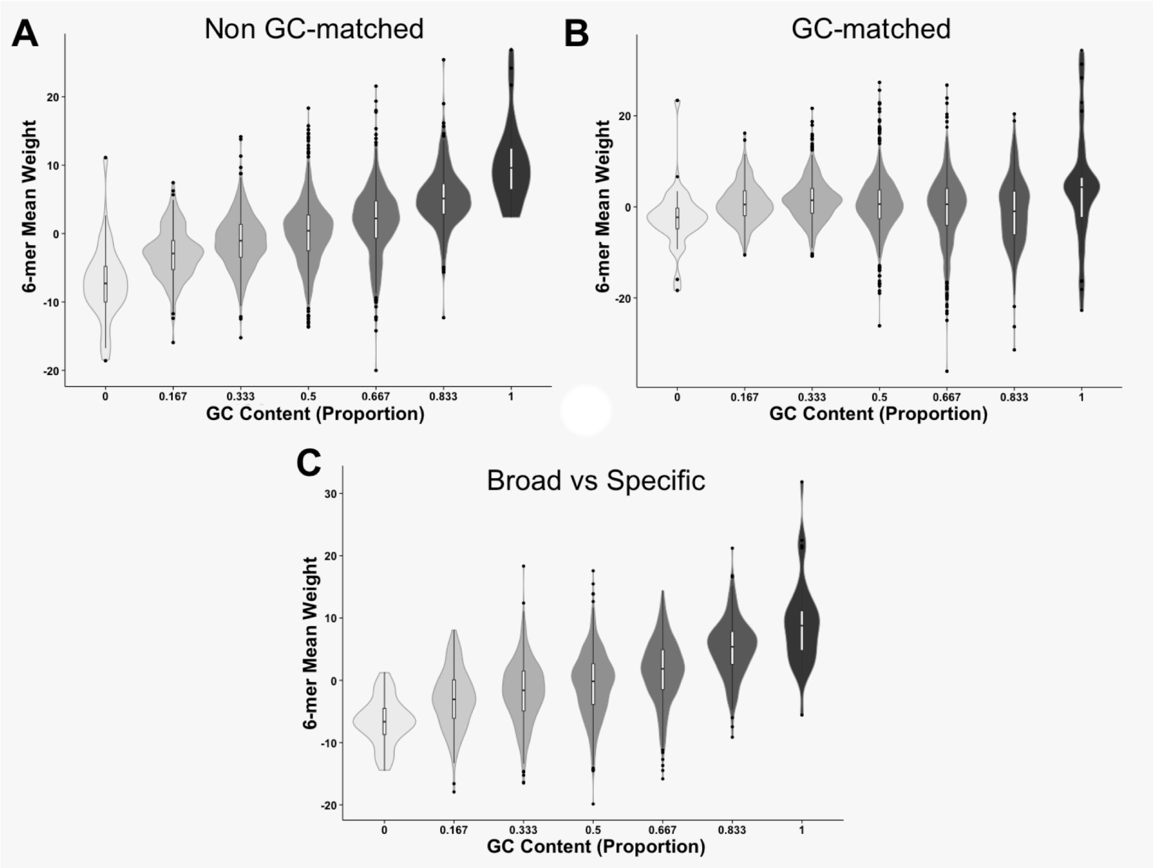

Figure S9. Short DNA sequence patterns predictive of broadly active enhancers have high GC content. The mean weight assigned by the SVM to each 6-mer is plotted against the GC-content of the 6-mer for (A) the non GC-matched model, (B) the GC-matched model, and (C) the context-specific model. Means were taken over the learned weights from four models trained on different negative sets.
